# The journey to antiretroviral therapy in Karnataka, India: who was lost on the road?

**DOI:** 10.7448/IAS.16.1.18502

**Published:** 2013-08-27

**Authors:** Suresh Shastri, Srinath Sathyanarayna, Sharath Burugina Nagaraja, Ajay MV Kumar, Bharat Rewari, Anthony D Harries, Rony Zachariah

**Affiliations:** 1 Karnataka State AIDS Prevention Society, Bangalore, India; 2 International Union Against Tuberculosis and Lung Disease (The Union), South-East Asia Regional Office, New Delhi, India; 3 Central TB Division, New Delhi, India; 4 National AIDS Control Organization, New Delhi, India; 5 Médecins sans Frontières (MSF), Medical Department (Operational Research), MSF-Brussels Operational Centre, Luxembourg

**Keywords:** operational research, ART centres, loss to follow-up, India

## Abstract

**Introduction:**

One important operational challenge facing antiretroviral treatment (ART) programmes in low- and middle-income countries is the loss to follow-up between diagnosis of human immunodeficiency virus (HIV) and initiation of ART. This is a major obstacle to achieving universal access to ART. This study from Karnataka, India, tried to measure such losses by determining the number of HIV-positive individuals diagnosed, the number of them reaching ART centres, the number initiated on ART and the reasons for non-initiation of ART.

**Methods:**

A review of records routinely maintained under the National AIDS Control Programme (NACP) was carried out in six districts of Karnataka. HIV-positive persons diagnosed during the months from January to June 2011 in 233 public HIV-testing sites were followed up until December 2011 based on the pre-ART registers. A chi-square test was used to assess statistical significance.

**Results:**

Of 2291 HIV-positive persons diagnosed (52% male; mean age of 35 years), 1829 (80%) reached ART centres. Of the latter, 1166 (64%) were eligible for ART, and 959 (82%) were initiated on treatment. Overall losses (attrition) on the road between HIV diagnosis and ART initiation were 669 (29%). Deaths, migration and not willing to go to the ART centres were cited as the main known reasons for not reaching ART centres. For ART-eligible individuals who did not initiate ART, the most common known reasons for non-initiation included dying before initiation of ART and not being willing to start ART.

**Conclusions:**

In a large state of India, eight in ten HIV-positive persons reached ART centres, and of those found ART eligible, 82% start treatment. Although this is an encouraging achievement, the programme needs to take further steps to improve the current performance by further reducing pre-ART attrition. We recommend online registering of diagnosed HIV-positive patients to track the patients more efficiently.

## Introduction

At the end of 2010, an estimated 34 million people were living with the human immunodeficiency virus (HIV) worldwide and 6.6 million were receiving life-saving antiretroviral treatment (ART) [1]. Despite scaling up ART in low- and middle-income countries, an estimated 9 million people eligible for ART did not have access – coverage has been 36% of those in need of ART. Universal access to treatment (defined as 80% or greater coverage) is thus still to be achieved in almost all parts of the world [1].

In India, the total number of people living with HIV/AIDS (PLHIV) was estimated at 2.4 million in 2009 [2]. In recent years, the country has put considerable efforts in expanding HIV-testing sites, and about 200,000 new HIV-positive individuals are diagnosed each year [2]. Access to CD4-testing facilities and ART centres has also been expanded. Despite such expansion, late presentation of patients to ART sites is known to be an operational challenge for two major reasons. One is failure to enrol for care after HIV diagnosis and retention after enrolment in pre-ART care; and the other is failure to initiate treatment promptly among those who are already ART eligible at the time of HIV diagnosis [3,4].

In the State of Karnataka in south India, HIV-testing sites and ART facilities have been scaled up. However, HIV-testing sites are physically disconnected from ART centres. While HIV-testing facilities are available at the primary healthcare level, ART centres are situated mostly at the district level. For an HIV-positive individual, this thus implies a “journey” from diagnosis to care that involves travel, time and other direct and indirect costs. This geographical disconnect between HIV-testing sites and ART centres might be resulting in pre-ART losses to follow-up (patients lost on the road to ART).

There is no published information on how well the linkage between HIV-testing sites and ART centres is functioning in India and on pre-ART losses to follow-up. Such information will be vital in improving the linkage between HIV testing and ART uptake among eligible individuals.

In the State of Karnataka, we thus determined pre-ART losses to follow-up. In particular, we determined (a) the number of HIV-positive persons diagnosed at HIV-testing sites, (b) the number who arrived at ART centres, (c) the reasons for non-arrival at ART centres, and (d) among those who did arrive, the number found eligible for and eventually initiated on ART, and reasons for non-initiation of ART.

## Methods

### Study design

This was a cross-sectional study involving a review of records routinely maintained under the National AIDS Control Programme (NACP).

### Study setting, sites and study population

Karnataka, with a population of 61 million, is one of the four largest states in south India and is considered to have a relatively advanced HIV epidemic. In 2009, the state had an HIV prevalence of 0.63%, amounting to 250,000 persons living with HIV [2].

There are 1615 public HIV-testing sites (565 are stand-alone, while 1050 are facility integrated) and 49 ART centres in Karnataka. All HIV-positive persons diagnosed at the HIV-testing sites are referred to the nearest ART centre for further management and are expected to reach these ART centres on their own. ART centres, as mentioned, are mostly located at the district level far from the point of HIV diagnosis; distances are in the range of 5–80 km, and they are often not well connected by public transport. Patients most often have to spend a whole day for each visit to the ART centre.

On arrival at ART centres, patients are registered, given a pre-ART registration number and classified into World Health Organization (WHO) clinical stages. They then undergo basic investigations, including CD4 count assessments, and if found ART eligible they are initiated on ART and followed up monthly. Those who are not eligible for ART are asked to return for six monthly follow-ups, including a CD4 assessment. ART eligibility assessments were done in accordance with national guidelines, which are in tune with current WHO recommendations. Briefly, any person in WHO clinical stages 1 or 2 with a CD4 count ≤350 cells/mm^3^ and anyone in stages 3 or 4 irrespective of CD4 count were eligible for ART.

Karnataka has 26 District AIDS Prevention and Control Units (DAPCUs) monitoring the operations under NACP. Each unit is headed by a district-level programme officer who in turn is assisted by paramedical staffs to monitor the process of linking HIV-positive persons who were diagnosed at HIV-testing sites to the ART centres, and to maintain information on all of the HIV-positive patients detected in their respective districts.

**Figure 1:**
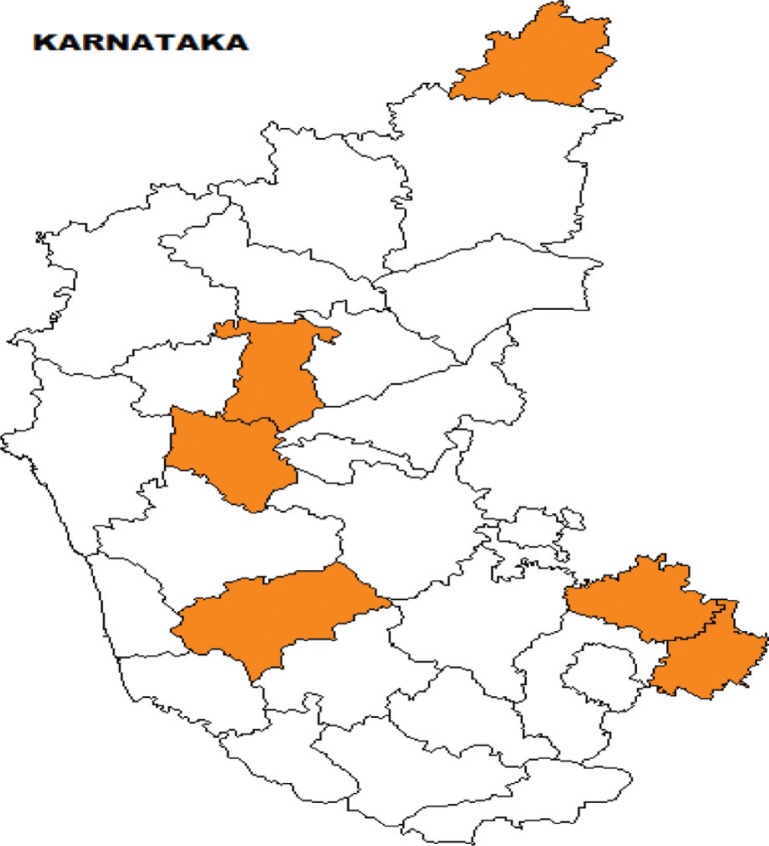
Map showing selected ART centres in the State of Karnataka, India.

This study was conducted at six districts of Karnataka (Figure 1), which were selected purposively from all of the four administrative zones of the state. In each of these districts, HIV-positive persons detected during the months of January to June 2011 were line-listed from all 233 public HIV-testing sites and assessed for the study objectives until the end of December 2011.

### Data and statistical analysis

The sources of data were HIV-testing records and ART centre records (pre-ART registers, ART enrolment registers and patient treatment cards). For each patient, we reviewed the records of respective districts through the end of December 2011 from the date of HIV diagnosis to ascertain whether the respective patient had registered or not. Pre-ART (HIV care) registers in the respective districts were reviewed until the end of December 2011 from the date of HIV testing to assess whether the patient had arrived at the ART centre. Patient details (name, age and sex) in the HIV-testing registers were matched with those in the pre-ART register to confirm the registration. If there was no matching on any of these variables, then the patient was designated a “loss to follow-up before registration for pre-ART.” In other words, all diagnosed HIV-positive patients who were not registered for pre-ART care even after 6 months were operationally defined for our study purpose as “loss to follow-up.” If the patient was not found registered in the ART records, then the information was sent to the respective DAPCU of the district and the concerned HIV-testing site where the diagnosis was made. The reasons for not presenting at ART centres were assessed by the staff of the DAPCU and the HIV-testing site by calling the telephone numbers of patients provided at the time of HIV testing or by visiting the patient's residence. Informed consent for home visits was taken at the time of HIV diagnosis and was done by either the HIV programme staff or representatives of local non-governmental organizations. The reasons were classified as death, patient refusal, inability to reach the ART centre due to social or economic constraints, migration out of the district, patient not contactable due to incomplete or incorrect address, patient being severely ill and bedridden or unknown reason. If it was found that the patient was registered for HIV care in another district, the same was noted and the patient was considered as having reached an ART centre.

For all patients who were registered in pre-ART, data were collected on whether they were assessed for eligibility for ART. If the patients were found eligible for ART, information on whether they were provided ART was collected.

About 10% of the HIV-testing sites were randomly selected, and the data manager of the ART centre and the district supervisor assessed the completeness and consistency of the data by verifying the records at the HIV-testing sites. Similarly, 10% of the overall patients line-listed were reviewed to assess whether the ART initiation status recorded or provided was accurate or not.

Data were double entered into a structured format created in EpiData statistical software, validated and analysed. Numbers and proportions as per study objectives were calculated. A chi-square test or Fischer's exact tests were used, as applicable, to compare proportions, and *p* ≤5% was considered statistically significant. Relative risks (RRs) and 95% confidence intervals (CIs) were calculated to measure the strength of association between exposures and outcome variables.

### Ethics approval

The study was approved by the Karnataka State AIDS Prevention Society and the Ethics Advisory Group of The International Union Against Tuberculosis and Lung Disease, Paris, France. According to the NACP guidelines, all patients attending voluntary counselling and the testing centre are counselled by professional-trained counsellors, and informed consent of the same was obtained from the patients.

## Results

### Attrition on the road between HIV diagnosis, ART eligibility and initiation

A total of 2291 HIV-positive persons were diagnosed in Karnataka, of which 1829 (80%) reached ART centres. About half of them were male, and the mean (SD) age was 35 (13) years. The median time taken for such patients to arrive at the ART centre was 4 days (range: 1–359 days). The characteristics of these individuals compared to those who did not reach the ART centres are shown in Table 1. There were no significant differences, by age or sex, in the proportions of patients reaching ART centres. All but one of 250 HIV-TB co-infected patients and all antenatal women (90) had reached ART centres. Among those who reached the centres and had their CD4 counts assessed, the median (interquartile range) CD4 count was 257 (116–478)/mm^3^.

**Table 1:** Characteristics of HIV-positive individuals who reached ART centres in Karnataka, India (January–June 2011)

	Total	Not reached		
				
	*n*	*n* (%)	RR (95% CI)	*p*
Total	2291	462 (20)		
Sex*				
Male	1201	237 (20)	1.02 (0.86–1.21)	0.84
Female	1071	207 (19)	Reference	
Age groups (in years)				
0–14	145	41 (28)	1.74 (0.80–3.79)	0.13
15–24	199	32 (16)	0.99 (0.45–2.20)	0.98
25–34	715	144 (20)	1.24 (0.59–2.62)	0.56
35–44	715	153 (21)	1.32 (0.63–2.78)	0.45
45–54	350	59 (16)	1.04 (0.48–2.24)	0.92
55–64	130	27 (21)	1.28 (0.57–2.87)	0.54
65 and above	37	6 (16)	Reference	
HIV-TB				
HIV without TB	2041	461 (23)	Reference	
HIV with TB	250	1 (0)	0.02 (0.00–0.13)	<0.01
Antenatal woman				
Antenatal woman	92	0 (0)	NA	<0.01
Rest	2199	462 (21)		

* There were 19 cases whose sex was not known.

ART= antiretroviral treatment; NA=not applicable; RR=relative risk; CI=confidence interval; TB=tuberculosis.

**Figure 2:**
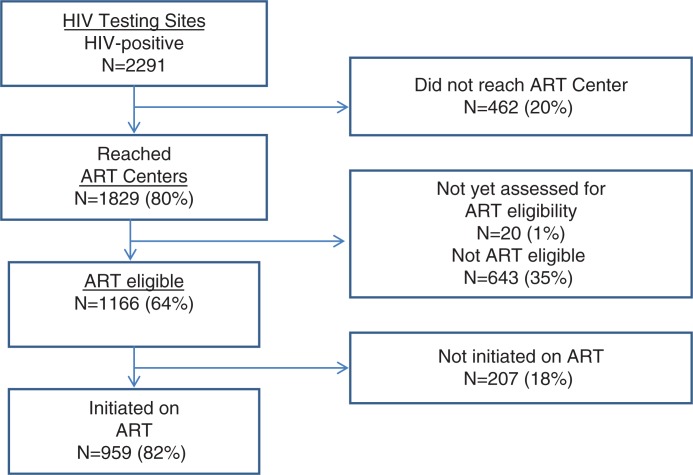
Pathway to antiretroviral treatment for HIV-positive individuals diagnosed in Karnataka, India between January and June 2011.

Of those who did manage to reach ART centres, 1166 (64%) were eligible for ART, of whom 959 (82%) were initiated on treatment. Median time delay between being found ART eligible and initiating treatment was 9 days (range: 2–325 days) (Figure 2). The characteristics of ART-eligible individuals not initiated on ART are given in Table 2. Compared to other sub-groups, higher proportions of persons in the age group of 55 years and above were not initiated on ART. The median (interquartile range) CD4 count among those eligible for ART was 145 (81–245)/mm^3^ and did not vary significantly among those who were initiated on ART (147, 81–238) and those who were not initiated on ART (141, 78–279) (*p*=0.8).

**Table 2:** Characteristics of ART-eligible individuals not initiated on treatment in Karnataka, India (January–June 2011)

	Total	Not initiated on ART		
				
Characteristics	*n*	*n* (%)	RR (95% CI)	*p*
Total	1166	207 (18)		
Sex				
Male	682	127 (19)	1.13 (0.87–1.45)	0.36
Female	484	80 (17)	Reference	
Age group (in years)				
0–14	52	2 (4)	Reference	
15–24	65	5 (8)	2.00 (0.40–9.89)	0.38
25–34	336	68 (20)	5.26 (1.33–20.82)	<0.01
35–44	403	71 (18)	4.58 (1.16–18.12)	<0.01
45–54	206	30 (15)	3.79 (0.93–15.33)	0.04
55–64	80	24 (30)	7.80 (1.92–31.62)	<0.001
≥ 65	24	7 (29)	7.58 (1.70–33.82)	<0.01
TB-HIV				
Without TB	947	166 (18)	Reference	
With TB	219	41 (19)	1.07 (0.78–1.45)	0.68
Antenatal care				
No	1131	203 (18)	Reference	
Yes	35	4 (11)	0.64 (0.25–1.61)	0.32

ART=antiretroviral treatment; RR=relative risk; CI=confidence interval.

Overall attrition on the road between HIV diagnosis and ART initiation was 669 (29%), (462 individuals were lost between HIV diagnosis and arrival at ART sites, and 207 were lost before ART initiation) (Figure 2).

### Reasons for losses on the road to ART (non-arrival at ART centres and non-initiation on ART)

The reasons for not reaching ART centres and for not being initiated on ART are shown in Table 3. Deaths, migration from the address given at the time of diagnosis and unwillingness to go to the ART centres were cited as the commonest known reasons for not reaching ART centres. For ART-eligible individuals who did not initiate ART, the most common known reasons for non-initiation included death before initiation of ART, not willing to get initiated on ART and transfer of the person to another district.

**Table 3:** Reasons for patients not reaching ART centres and not initiating ART in Karnataka, India (January–June 2011)

Reasons for not reaching ART centres (*n*=462)^a^	*n* (%)
Death	121 (39)
Migration	66 (21)
Not willing	53 (17)
Incorrect address	20 (6)
Out-of-district patient – no information	38 (12)
Others^b^	16 (5)
Not known^c^	148
**Reasons for not initiating ART in eligible patients (*n=*207)^a^**	
Death	57 (43)
Not willing	20 (15)
Transferred out	16 (12)
Others^b^	40 (30)
Not known^c^	74

a Only one reason was recorded per individual. ART=antiretroviral treatment.

b Others: social reasons, economic reasons, taking treatment in private sector, or bed ridden or seriously ill.

c These patients could not be contacted and are excluded from the denominator while calculating percentages.

## Discussion

This is one of the first studies in India that assessed the effectiveness of the linkage between HIV-testing sites and ART centres, and it shows that about one in three HIV-positive individuals are lost along the way to receiving ART. The most common known reason was early death.

A key finding of this study is that, in the “linkage pathway” between HIV diagnosis and eventual ART initiation, 71% of HIV-positive individuals were retained in care and 82% of those who were ART eligible accessed treatment. A recent systematic review estimated that less than a third of patients testing HIV-positive in sub-Saharan Africa remained continuously in pre-ART care until ART initiation [5]. In this respect, our results are better than the global average [1] and those reported from the sub-Saharan regions. This notwithstanding, we feel that an overall figure of 29% attrition is still too high as it risks challenging the credibility of the ART programme in the view of patients, health workers and the community at large. *What can be done to improve the situation?*

First, since over 26% of attrition was related to early deaths at the stages of HIV testing and ART eligibility, it is likely to reflect late presentation of patients and advanced disease. A recent study from India showed that 85% of patients present late to health facilities [6]. A triage system is thus needed both at HIV-testing sites and ART centres which will allow identification and fast-tracking of individuals presenting lately. There might also be a need to provide support for referral for very ill patients through provision of transport and other social support measures so that they can arrive and start ART earlier. The fact that one in three vulnerable ART-eligible individuals over 55 years of age did not start treatment tends to support this recommendation.

Second, the high proportion of early deaths also supports the 2011 Indian government decision to raise the CD4 count threshold for ART initiation from 250 to 350 cells/mm^3^ as this will tend to foster a “health cohort effect” and facilitate ART initiation before patients reach an advanced stage of disease.

Third, there is a clear need to decentralize ART further and bridge the geographical disconnect between HIV testing and care, as separate (disconnected) services are known to result in lower ART uptake [7].

Fourth, as is done in tuberculosis programmes, all HIV-positive individuals once diagnosed at HIV-testing sites should receive a unique HIV registration number which they continue to use during ART initiation and beyond. This is not the situation at present. It is well known that the greatest difficulty in tracing lost HIV-positive individuals is found after HIV diagnosis and before they reach ART centres [8]. Ensuring a unique registration number at HIV diagnosis will allow both cohort monitoring and tracing of patients [3]. As India is moving towards an online electronic system that will include all HIV-positive individuals and those who start ART, there is ample opportunity to use this opportunity to start a unique registration number system.

A very encouraging finding from this is that almost all (i.e. all but one) co-infected HIV-TB patient and all HIV-positive pregnant women reached ART centres which is very encouraging as compared to results of studies elsewhere [7,9]. This reflects the fruits of joint operational efforts led by both NACP and the Revised National Tuberculosis Control Programme (RNTCP) to improve joint TB-HIV management in recent years. This involved intensified training and follow-up of HIV-infected TB patients in the community. This experience could perhaps show the way forward for other groups of patients who have not yet benefitted in a similar manner.

Finally, in 222 HIV-positive individuals who were lost to follow-up, the reasons were unknown despite efforts to trace them. This merits further investigation.

The strengths of this study are that patients were rigorously traced between HIV-testing sites and ART centres, data were double entered and validated, we tried to explore reasons for losses to follow-up through telephone calls and outreach visits and, as the data come from routine clinics, it is likely to reflect the on-the-ground reality. We also adhered to the guidelines for reporting of observational studies [10] and ethics [11]. Limitations include the fact that in roughly 35% of patients who were lost to follow-up, we were unable to ascertain the reasons, and as the data come from six purposively sampled districts there may be limits on its wider generalizability.

In conclusion, in a large state of India, 80% of all HIV-positive persons reach ART centres, and of those found ART eligible, 82% start treatment. Despite this encouraging achievement, the programme is taking further steps to improve the current performance by trying to further reduce attrition on the way to ART.
